# Culturalizing theory and research on cognitive models of hope

**DOI:** 10.3389/fpsyg.2024.1457725

**Published:** 2024-08-09

**Authors:** Allan B. I. Bernardo, Sixtus Dane A. Ramos

**Affiliations:** Department of Psychology, De La Salle University, Manila, Philippines

**Keywords:** hope, culture, cross-cultural, locus-of-hope, cognitive theories of hope

## Introduction

The study of hope has a long history across different disciplines; in psychology, cognitive theories of hope have driven much of the recent empirical research on the positive outcomes of hope (Rand and Rogers, 2023). Based on Snyder's (1994) theory, hope is defined as the disposition to identify goals, determine strategies, and muster the willingness to pursue these goals. In the last three decades, psychology research has accumulated evidence on hope's association with wellbeing (Pleeging et al., 2019) and on the benefits of hope interventions (Weis and Speridakos, 2011). However, conceptualizing hope as a cognitive disposition tends to overlook the social (interpersonal, normative) and cultural dimensions of hope. In this opinion, we assert that research on cognitive theories of hope needs to better understand cultural aspects of the cognitive disposition of hope. Below, we reflect on the universality of hope, current findings on hope across cultures, and prospects for stronger cultural theorizing on cognitive hope.

## Implicit universality in cognitive hope theory

The cognitive theory of hope assumes that people's behaviors are goal-directed, and they appraise their capacity to pursue these goals (Snyder, 2002). Drawing from theories of future-orientation, self-efficacy, personal mastery, among others, hope theory assumes three components of hope: goals that serve as targets of mental processes, pathways thinking (or strategies produced to attain goals), and agency thinking (or the perceived capacity to pursue goals despite challenges). Characterizing hope as a cognitive disposition may imply that the construct and theory are universal, but the theory did not explicitly state so. Snyder (1994) suggested that the goals, pathways, and agency dimensions of hope might not be equally expressed across different groups of people, considering some societal forces that may undermine the goal pursuit of particular groups. But Snyder did not suggest that the hope-related cognitive processes vary across cultures.

Indeed, research on hope theory seems to be motivated to show its universality. Studies that translate and/or tested the validity of the Dispositional Hope Scale (Snyder et al., 1991) in different cultures (Hellman et al., 2013; Edwards and McConnell, 2023) and evidence for invariance leads some to proclaim the scale's and theory's universality (Li et al., 2018). There are also references to consistent positive correlates of hope and positive effects of hope interventions across cultures, although most meta-analyses did not actually include culture, ethnicity, or country as moderators (Weis and Speridakos, 2011; Marques et al., 2017; Corrigan and Schutte, 2023). One meta-analysis looked at country location as a moderator (Reichard et al., 2013) and found stronger effect sizes in studies conducted in the USA compared to other countries.

## Cultural lenses of hope

Some scholars have elaborated on cultural dimensions of the experience of hope, and these expositions typically refer to affective and discursive dimension of hope (e.g., Crapanzano, 2003; Averill and Sundararajan, 2005). Attempts to consider culture in cognitive hope theories initially involved the imposed *etic* approach (Berry, 1989) to cross-cultural comparisons among cultural or ethnic groups within the USA (Chang and Banks, 2007; Hirsch et al., 2012). The *etic* comparisons were motivated by Snyder's (2002) hypothesis that encountering systemic obstacles in goal pursuit are likely to lower hope for some groups. Results did not always support the hypothesis and the group differences were discussed with some tentative reference to cultural factors.

### Divergent findings in diverse cultural contexts

The need to pay more careful attention to how culture relates to hope became more apparent when empirical research on cognitive theories of hope produced results that were not aligned with the theory. Research on translations and/or adaptations of the Dispositional Hope Scale did not always support the scale's two-factor structure (e.g., Brouwer et al., 2008; Pacico et al., 2013; Galiana et al., 2015; Savahi et al., 2016; Lei et al., 2019; Khumalo and Guse, 2022), suggesting that the fundamental distinction between hope pathways and agency may not hold in all cultures.

Correlates of hope were also not consistent across different cultural/ethnic groups. Chang and Banks (2007) found that the positive factor that most strongly predicted hope varied among different ethnic groups in the USA. For example, the strongest predictors of agentic thinking was life satisfaction for European Americans, but was negative problem orientation, rational problem solving, and positive affect for African American, Latinos, and Asian Americans, respectively. Moreover, hope did not have the expected buffering role in the relationship between race-related stress and wellbeing of African-Americans (McDermott et al., 2020). Outside North America, research also found cultural variations on the relationship between hope and negative affect (Hutz et al., 2014), resilience (Alali, 2020), mental health (Slezackova et al., 2023), and flourishing (Flores-Lucas et al., 2023).

Although hope interventions are shown to boost hope-related outcomes (Weis and Speridakos, 2011), there are inconsistencies. Indonesian survivors of a natural disaster (Retnowati et al., 2015) and a community sample from the United States (Cheavens et al., 2006) showed significant decrease in depression and anxiety after undergoing a hope intervention. A hope program for Portuguese middle-school students improved hope levels but not mental health outcomes (Marques et al., 2011). Hope interventions for cancer patients from China (Chan et al., 2019) and Korea (Shin and Park, 2007; Kim et al., 2008) produced inconsistent outcomes on hope, quality of life, and depression scores.

These cultural differences may be attributed to methodological factors, but they also imply cultural-level factors that should be considered in better understanding the psychological process that are assumed to be associated with hope.

### Cultural meanings of hope

Emic approaches (Berry, 1989) for studying hope in particular cultures can help make sense of cultural variations in hope. For example, spiritual beliefs and religious practices were mentioned as sources of hope among Latino families caring for a member with schizophrenia (Hernandez et al., 2019) and Filipino families caring for terminally ill adolescents (Briones and Bernardo, 2016). Family-relational processes were often mentioned in conceptions of hope in children in rural South Africa (Cherrington, 2018), adults from Ghana and South Africa (Wilson et al., 2021), Turkish teachers (Eren and Yesilbursa, 2017), Israeli families of children with special needs (Al-Yagon and Margalit, 2017), and refugees (Umer and Elliot, 2021). A study of disenfranchised young people in Australia indicates how minimal opportunity structures in their environment constrain how they define goals when thinking about the future (Bryant and Elland, 2015). Such emic studies on meanings and conceptions of hope indicate social or cultural sources of variations in the psychological mechanisms that underlie individuals' hope cognitions in different cultures.

### Cultural models of agency, pathways, and goals

There have been efforts to extend the cognitive theories of hope to accommodate specific cultural experiences. For example, using a bottom-up approach to defining hope factors among Taiwanese adults, Luo et al. (2010) added two scales (transcendental adaptation, persisting effort) to the Dispositional Hope Scale. Maree et al. (2008) developed a five-factor scale (goal achievement resources, ineffectuality, future vision, despondency, and self-efficacy) of South African hope derived from qualitative analysis of students' expressions of hope. Bernardo (2010) noted that hope agency and pathways in cognitive theories of hope assume disjoint agency toward goals pursuit and excludes conjoint agency that is important in collectivist cultures. He differentiated between internal (disjoint) and external (conjoint) locus-of-hope, and added external-family, external-peer, and external spiritual loci-of-hope. Different versions of the Locus-of-hope Scale were validated (Bernardo and Mendoza, 2021; Bernardo et al., 2022a), and evidence for the positive outcomes of external loci-of-hope have been found across different Asian (Bernardo et al., 2018b, 2022b; Tee et al., 2022) and North American (Munoz et al., 2019; Wagshul, 2019; Dargan et al., 2021) studies, including some showing its role in protecting individuals against negative mental health outcomes during the COVID-19 pandemic (Zhang et al., 2021; Dizon et al., 2023). Recently, a locus-of-hope intervention was shown to boost hope and recovery outcomes of Filipinos with substance use disorders (Ramos, 2023).

The above innovations still involved processes related to the pursuit of individual goals. Others hope scholars (Braithwaite, 2004) proposed that individuals may pursue goals that may involve other people, groups, and even whole societies, calling for a distinction between personal and collective hope (Sagy and Adwan, 2006). Jin and Kim (2019) also developed a Social Hope Scale to measure hope thinking that refers to collective goals.

## Toward cultural cognitive theories of hope

These recent developments point to significant efforts to consider cultural processes in constructing specific facets of cognitive hope theories. But when these cultural versions of hope theories find cultural differences (e.g., Du et al., 2015; Bernardo et al., 2018a), the differences are still attributed *post hoc* to some vague cultural factors. There is a need for more complete cultural cognitive hope theories that will explain cultural variations within the culture-adapted hope measures.

There are recent proposals that sketch components of a cultural theory of hope. Krafft et al.'s (2023b) theory is perhaps the most comprehensive proposal that has as its core the components of cognitive theories of hope, but the theory has other important propositions that can account for cultural specificities. First, the theory constructs the goals or hoped-for ends as possibly being both individual and collective, and that are likely to be influenced by culturally transmitted values and norms (Krafft et al., 2023d). Second, the theory assumes that hope involves the belief in the possibility of the fulfillment of the goals. Finally, the theory adds the component of trust in the resources, both internal and external, for achieving the goals. The last two components are shaped by social experiences, norms and cultural belief systems as indicated by some cross-cultural studies (Bernardo and Nalipay, 2016; Krafft et al., 2023a,c).

Krafft et al.'s (2023a) proposal identifies the pathways for how different cultural-level factors (e.g., values, beliefs, etc.) bear on the cognitive-motivational processes underlying the hope experience of particular societies/cultures. It might still be necessary to identify specific values, beliefs, social norms, and others cultural sources that would moderate the cognitive-motivational hope mechanisms. But the theoretical proposals allow for more precise hypotheses about cultural differences in hope levels based on assumptions about culture-level differences in the antecedent factors. More interesting predictions would explain differences in the correlates and consequences of hope, and even in how hope moderates other psychological processes in particular cultural groups. Indeed, it may even be possible to predict when hope leads to non-positive outcomes in particular groups within a particular social and cultural milieu. It may also provide a better understanding of and guide for how hope interventions enhance wellbeing across groups by pointing researchers to target specific change mechanisms suitable to the particular cultural processes to promote future thinking and better mental health.

We recognize that these proposals to “culturalize” cognitive theories of hope are aligned with the so-called third-wave of positive psychology (Lomas et al., 2021), which involves shifting from a primary focus on individuals to understanding how positive psychological processes in individuals are embedded in groups and systems, and are therefore, cultural. The proposals are also consistent with Wissing's (2022) proposals for a post-disciplinary approach to wellbeing research that emphasizes contexts and meta-theoretical assumptions or worldviews. As with other positive psychological and wellbeing concepts, there is a growing consensus that these positive processes do not reside only within individuals, and that individuals' positive functioning is dynamically linked with affordances, constraints, and meaning systems in their sociocultural environments.

We should note that the preceding proposals (Lomas et al., 2021; Wissing, 2022) also emphasized using more varied methodologies. We acknowledge that the rapid growth of empirical research on cognitive theories of hope since the 1990s was driven in part by the availability of the short, easy-to-use self-report measure (Snyder et al., 1991). But if we aim to build knowledge about the psychology of hope that will resonate with the diverse experiences of individuals in different cultures, we will need to develop more multidimensional measures of hope, gather more diverse forms of quantitative and qualitative data, use rigorous frameworks for developing and testing the efficacy of hope interventions across cultures, and apply analytic approaches that are attuned to how people in diverse cultures express and experience hope.

## Conclusion

In the Philippines, there is a saying, “*Habang may buhay, may pag-asa*;” in China there is a proverb that says, “留得青山在, 不怕没柴烧。” Both sayings may be understood in English as, “While there is life there is hope.” Indeed, the idea that hope is an essential part of life is probably found in many cultures (Bishop and Willis, 2014). But hope is understood and experienced in distinctive ways in different cultures. A valid psychological theory of hope should aim to capture the diversity in cultural experiences of hope, as it reveals those aspects of hope shared by all humans. We contend that there are now viable pathways toward a culturalized cognitive theory of hope.

## Author contributions

AB: Conceptualization, Formal analysis, Supervision, Writing – original draft. SR: Conceptualization, Formal analysis, Writing – original draft.
